# CDK4/6 inhibitor-SHR6390 exerts potent antitumor activity in esophageal squamous cell carcinoma by inhibiting phosphorylated Rb and inducing G1 cell cycle arrest

**DOI:** 10.1186/s12967-017-1231-7

**Published:** 2017-06-02

**Authors:** Jiayuan Wang, Qingqing Li, Jiajia Yuan, Jingyuan Wang, Zuhua Chen, Zhentao Liu, Zhongwu Li, Yumei Lai, Jing Gao, Lin Shen

**Affiliations:** 1 Department of Gastrointestinal Oncology, Key Laboratory of Carcinogenesis and Translational Research (Ministry of Education/Beijing), Peking University Cancer Hospital and Institute, Fu-Cheng Road 52, Hai-Dian District Beijing, 100142 China; 2 Department of Pathology, Key Laboratory of Carcinogenesis and Translational Research (Ministry of Education/Beijing), Peking University Cancer Hospital and Institute, Fu-Cheng Road 52, Hai-Dian District Beijing, 100142 China

**Keywords:** CDK4/6 inhibitor, ESCC, Cell cycle arrest, pRb

## Abstract

**Background:**

Cell cycle dysregulation is common in human malignancies, and CDK4/6 inhibitors targeting cell cycle have potential antitumor activity. SHR6390 is a novel small molecule inhibitor specifically targeting the CDK4/6 pathway. However, the role of SHR6390 in esophageal squamous cell carcinoma (ESCC) remains unknown, which will be investigated in our study.

**Methods:**

Eca 109, Eca 9706, and KYSE-510 ESCC cell lines were chosen for further analysis. The effect of SHR6390 on cell viability, cell cycle and cell apoptosis, the status of kinases in Cyclin D1-CDK4/6-Rb pathway were determined by MTS assay, flow cytometry, and western blotting, respectively. Cell-derived and patient-derived xenografts were established to investigate the effects of drugs in vivo.

**Results:**

SHR6390 could suppress cell proliferation in vitro cell lines and inhibit tumor growth in vivo PDX models with different drug susceptibility. The effective treatment of SHR6390 induced the inhibition of phosphorylated Rb and cell cycle arrest at G1 phase both in cell lines and in xenografts. SHR6390 combined with paclitaxel or cisplatin offered synergistic inhibitory effects in cell-derived xenografts especially in Eca 9706 xenografts which showed relative lower sensitivity of SHR6390 single. Moreover, low expression of CDK6 and/or high expression of Cyclin D1 might be associated with high sensitivity of SHR6390, which would be validated in the future.

**Conclusions:**

CDK4/6 inhibitor-SHR6390 exerted potential antitumor activity against ESCC cell lines and xenografts, and evaluation of CDK6 and Cyclin D1 expressions might be helpful to select patients beneficial from SHR6390, which provided evidences for future clinical trials.

**Electronic supplementary material:**

The online version of this article (doi:10.1186/s12967-017-1231-7) contains supplementary material, which is available to authorized users.

## Background

Esophageal cancer (EC) is the leading cause of cancer mortality in China [1]. The most common variant of esophageal cancer prevalent in China is esophageal squamous cell carcinoma (ESCC). Current therapies are concentrated on surgery, chemotherapy and radiotherapy, which offer poor prognosis with 5-year survival rate less than 20% [1, 2]. Thus, targeted-therapy based on genetic alterations may give promise.

Cell cycle dysregulation indicated by abnormal expressions and variations (mutations, amplifications, and deletions) were noted to occur frequently in human malignancies [3, 4]. Given its importance in cell cycle control, Cyclin D1-CDK4/6-Rb pathway is a highly validated anticancer drug target [5]. Early in the G1 phase of cell cycle, Cyclin D1 activates CDK4/6, and phosphorylates Rb subsequently. Phosphorylation of Rb reduces the inhibitory control of the transcription factor E2F, which enables the cell to pass through the G1 restriction point into S-phase [6]. Deregulation of the Cyclin D1-CDK4/6-Rb pathway triggered loss of cell-cycle control, one of the hallmark of cancer inducing carcinogenesis [7]. Targeting CDK4/6 mediated Rb phosphorylation by small molecule inhibitors has the possibility to block cell cycle progression and suppress tumor growth [8]. CDK4/6 has proven to be an effective target in diseases spanning breast cancer to colon cancer and neuroblastoma [9–13]. CDK4/6 inhibitor has been granted FDA approval as breakthrough therapy of breast cancer. Genomic characterization has demonstrated that ESCC harbour amplification of CDK6 and Cyclin D1, deletion of p16, and mutations of Rb, which are important regulators of cell cycle [14]. This suggests the potential utility of CDK4/6 inhibitors in ESCC.

Here, we aimed to evaluate the anti-tumor activity of SHR6390, which is an orally bioavailable, small molecule CDK4/6 inhibitor, in ESCC in vitro cell lines and in vivo PDXs models. Moreover, we investigated the possible mechanisms of SHR6390 and the effects of SHR6390 combined with paclitaxel (PTX) or cisplatin (CDDP). Finally, we sought to identify response markers known to be implicated in Cyclin D1-Rb-CDK4/6 signaling. This study will provide direct evidences for the future clinical trials.

## Methods

### Cell lines and reagents

ESCC cell lines Eca 109, Eca 9706 and KYSE-510 were obtained from the Cell Bank of the Peking Union Medical College (Beijing, China). The cells were cultured in RPMI-1640 media (Gibco-BRL, MD, USA) supplemented with 10% fetal bovine serum (FBS; Gibco-BRL) and 1% penicillin and streptomycin (Gibco-BRL) in a humidified incubator (37  °C) with 5% CO_2_. The CDK4/6 inhibitor SHR6390 (purity  ≥99%) which is a selective small-molecular CDK4/6 inhibitor was kindly provided by Jiangsu Hengrui Medicine Co., Ltd (Jiangsu, China). Paclitaxel (PTX) (purity  ≥ 99.9%) was purchased from Beijing Union Pharmaceutical Factory (Beijing, China), and cisplatin (CDDP) (purity  ≥ 99.9%) was purchased from Hospira Australia Pty Ltd (Australia). For in vitro studies, SHR6390 was dissolved in dimethyl sulfoxide at a stock concentration of 10 mmol/L and stored at −20 °C until further use.

### Cell viability assay

Eca 109, Eca 9706 or KYSE-510 cells were seeded into 96-well plates at a density of 3–5  ×  10^3^ cells/well overnight. Cells were treated the next day with SHR6390 for 72  h, and then assessed for viability using the MTS assay (CellTiter 96 Aqueous One Solution Cell Proliferation Assay, Promega, Madison, WI, USA) according to the manufacturer’s instructions. The absorbance was measured at 490 nm using a spectrophotometer. All experiments were repeated and read three times for each concentration.

### RNA interference

The siRNAs targeting CDK6 and Cyclin D1 were purchased from RiBoBio Co., Ltd (Guangzhou, China), which transfected into Eca 109 or Eca 9706 cells using the Lipofectamine^®^ 3000 reagent (Effectene, Qiagen, USA) according to the manufacturer’s protocol. At 24 h after transfections, cells were cultured in RPMI-1640 medium supplemented with 10% FBS (Gibco-BRL) for another 24 h before using for other experiments.

### Western blots

Eca 109, Eca 9706, and KYSE-510 cells were starved in serum-free medium overnight, and SHR6390 were added for 24 h. The Cell pellets and tumor tissues of xenografts were lysed using RIPA Lysis Buffer (Beyotime Biotechnology, Jiangsu, China) on ice, containing complete protease inhibitor and phosphatase inhibitor cocktail (Roche, Switzerland). Protein concentrations were measured using the BCA Protein Assay Kit (Beyotime Biotechnology, Jiangsu, China). Protein samples were diluted to equal concentrations (40 μg), and separated by electrophoresis in 10–12% SDS-PAGE and transferred onto nitrocellulose membranes (GE Healthcare, Piscataway, NJ). Antibodies used were against: CDK6 (sc-177) (Santa Cruz Biotechnology, Santa Cruz, CA, USA); Rb(#9313S), pRb(#9307S), CDK4(#12790), Cyclin D1 (#2978) (Cell Signaling Technology, Boston, MA, USA); β-actin (#014M4759) (Sigma-Aldrich, USA). All antibody except CDK6 (1:100) dilutions were 1:1000. Proteins were visualized using ECL plus Western Blotting Detection Reagents (GE Healthcare). Densitometry analysis of the Western blot protein was performed using the ImageJ software.

### Cell cycle assay

After treated with SHR6390 for 24 h, cell pellets were harvested and fixed in 70% cold ethanol overnight at 4 °C. Fixed cells were stained with 50 μg/mL propidium iodide (BD Biosciences), and incubated for 30 min at room temperature in the dark. Cell cycle analysis was performed using a FACS Calibur system (BD Biosciences). Data were analyzed by ModFit 3.0 software (BD Biosciences).

### Annexin V apoptosis assay

Cells were exposed to SHR6390 for 24 h and next were conducted by staining with Annexin V-Allophycocyanin (APC) and 7-amino-actinomycin (7-AAD) (BD Biosciences, Erembodegem, Belgium) for 15 min at room temperature in the dark, followed by flow cytometric analysis within 1 h (BD Biosciences). Cell apoptosis was analyzed by using the WinMDI 2.9 software (BD Biosciences).

### Xenograft models in immunodeficiency (NOD/SCID) mice

Two kinds of xenograft models were used in our study. Eca 109 cells and Eca 9706 (1–2  × 10^6^) cells were suspended in 100 μL of phosphate-buffered saline (PBS), and injected subcutaneously into the flanks of 6-week-old female NOD/SCID mice (Beijing HFK Bio-Technology Co., LTD, Beijing, China). Six patient-derived xenografts (PDX) were obtained according to a previously published report [15]. Tumors were measured with fine calipers every 2 days. When tumors was about 150–200 mm^3^, mice were randomized into six groups (5–6 per group) and treated with saline; SHR6390 (150 mg/kg weekly, oral gavage); PTX (3 mg/kg twice weekly, ip); or CDDP (3 mg/kg twice weekly, ip); SHR6390 and CDDP or PTX (see previous doses). Animals were treated for 3 weeks. Calculations in our study were used as follows:

$$ {\text{Tumor volume }} =  {{\left( {{\text{Length }} \times {\text{ Width}}^{2} } \right)}/ 2} $$

$$ {\text{Tumor growth inhibition }}\left( {\text{TGI}} \right)  = \Delta {\text{T}}/\Delta {\text{C }} \times 100\% $$

$$\begin{aligned} {\text{Tumor regression rate }}\left( {\text{TRR}} \right)  & =  {{\left( {{\text{V}}_{\text{pre}} {-}{\text{ V}}_{\text{post}} } \right)} / {{\text{V}}_{\text{pre}} }} \\ & \quad \times  100\% \end{aligned}$$

ΔT = tumor volume change of the drug-treated group on the final day of the study, ΔC = tumor volume change of the control group on the final day of the study, V_pre_ = pre-drug tumor volume, V_post_ = post-drug tumor volume.

The collection of tissue samples was approved and supervised by the Research Ethics Committee of Peking University Cancer Hospital & Institute. All patients signed written informed consent for their samples to be used in the study. All animal experiments were performed in accordance with the animal experimental guidelines of Peking University Cancer Hospital and followed internationally recognized ARRIVE (Animal Research: Reporting of In Vivo Experiments) guidelines.

### Statistical analysis

Statistical analysis was performed with SPSS 20.0 software. For in vitro studies, differences between groups were conducted by one-way ANOVA, unpaired two-tailed t test or factorial analysis. For in vivo studies, tumor growth in different groups was compared using repeated measures ANOVA. *P* < 0.05 was considered statistically significant.

## Results

### CDK4/6 inhibitor SHR6390 strongly inhibited tumor proliferation of ESCC in vitro and in vivo

**Fig. 1:**
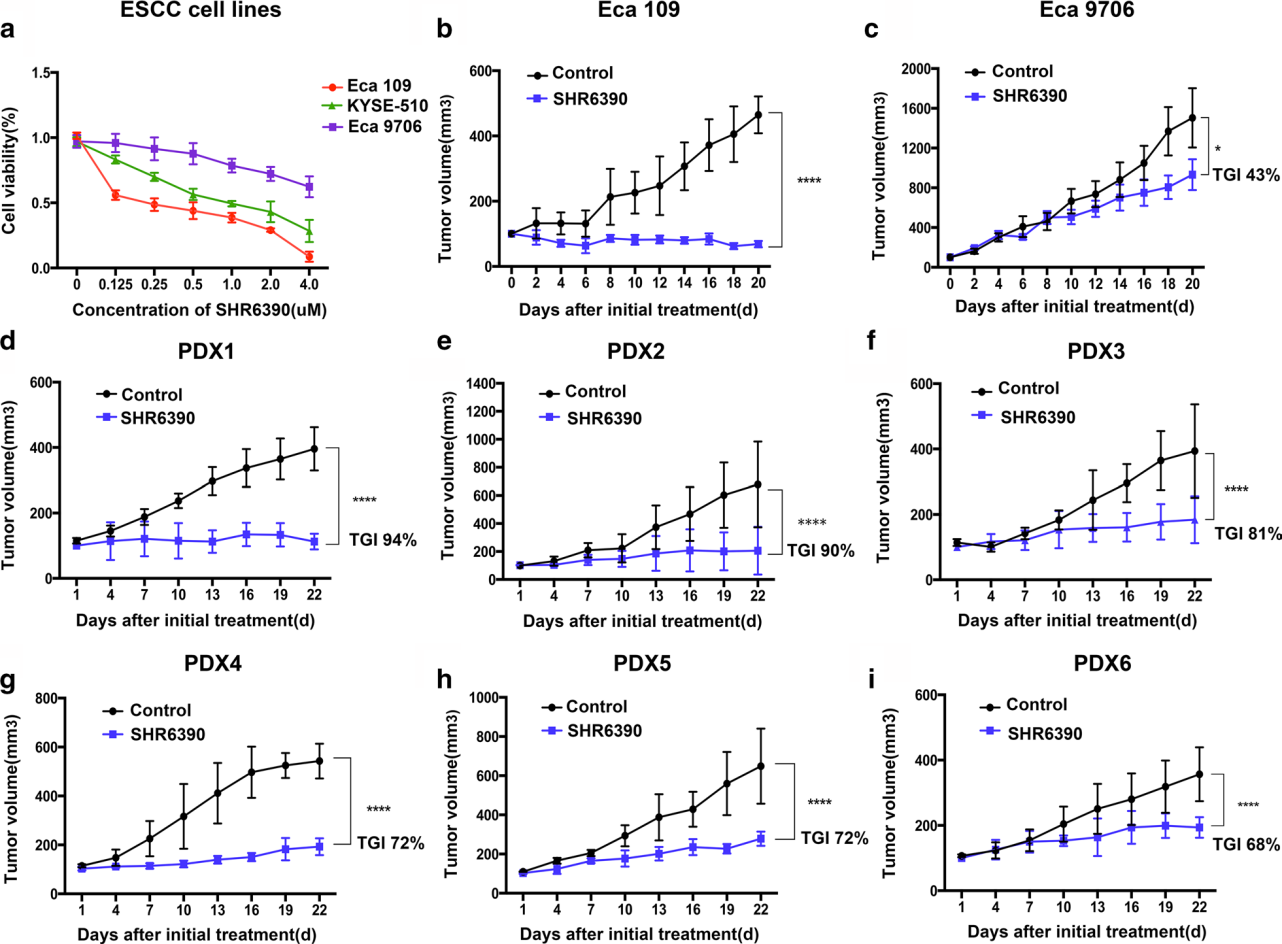
Antitumor effects of SHR6390 in ESCC cell lines and xenografts. **a** SHR6390 showed inhibitory activity against ESCC cells with different drug susceptibility. Proliferation assay are expressed as the mean ± SD of three replicate assays. **b** In vivo antitumor activity of SHR6390 in subcutaneous Eca 109 cells line-derived xenografts. SHR6390 caused tumor regression in Eca 109 xenograft. **c** In vivo antitumor activity of SHR6390 in subcutaneous Eca 9706 cell line-derived xenografts. The growth of tumors treated with SHR6390 alone was significantly suppressed in the Eca 9706 xenograft. **d**–**i** Effects of SHR6390 on the ESCC PDXs models. Tumors subcutaneously engrafted and grown until 150–200 mm^3^. Then, mice were treated with 150 mg/kg SHR6390 for 21 days and tumors were measured twice daily. Compared to the control group, the growth of tumor was significantly suppressed in SHR6390 treatment group with various suppressions. Tumor volume was expressed as mean ± SD. The antitumor activity is depicted by % TGI. TGI = ΔT/ΔC × 100% (ΔT = tumor volume change of the drug-treated group, ΔC = tumor volume change of the control group on the final day of the study). *P < 0.05, **P < 0.01, ***P < 0.001, ****P < 0.0001 by one-way ANOVA or unpaired two-tailed t test

The antitumor effects of SHR6390 were firstly assessed in Eca 109, Eca 9706 and KYSE-510 cell lines. As shown in Fig. 1a, SHR6390 inhibited cell proliferation in a dose-dependent manner, with Eca 109 being the relative sensitive one and Eca 9706 being the relative resistant one (Fig. 1a). Next we chose Eca 109 and Eca 9706 cell lines which responded differently to SHR6390 to established xenografts. The growth of tumors treated with SHR6390 alone was significantly suppressed both in Eca 109 and Eca 9706 xenografts compared to the control groups (Fig. 1b, c). Consistent with the results of cell lines, compared to Eca 109 derived xenografts, Eca 9706 derived xenografts showed relative lower sensitivity of SHR6390. Thus, SHR6390 restricts proliferation in ESCC cell lines and xenografts. The best preclinical PDX models were used in this study to further evaluate the antitumor activity of SHR6390 in vivo. Biopsies from ESCC patients were subcutaneously inoculated into NOD/SCID mice to establish ESCC PDXs models. A total of six PDX models were treated with control and SHR6390 for 3 weeks. Compared to the control group, the growth of tumor was significantly suppressed in SHR6390 treatment group (P  < 0.05 for all) (Fig. 1d–i). Meanwhile, we found a variable effect of SHR6390 on the different ESCC PDXs (68–94% TGI). Thus, SHR6390 inhibited proliferation of ESCC in vitro cell lines and tumor growth in vivo with various suppressions.

### SHR6390 induced the inhibition of Rb phosphorylation in vitro and in vivo

**Fig. 2:**
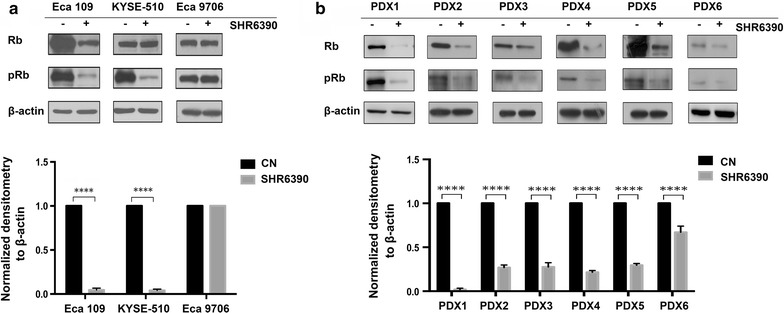
SHR6390 reduced Rb phosphorylation in vitro and in vivo. **a** Eca 109, KYSE-510 and Eca 9706 cells were treated with SHR6390 for 24 h, and then subjected to Western blotting. SHR6390 significantly blocks phosphorylation of Rb at serine 780 in relative sensitive Eca 109 and KYSE-510 cell lines, but not in relative resistant Eca 9706 cell line. **b** Tumor specimens of six ESCC PDXs were harvested after the end of treatment followed by analysis of lysates for Rb, pRb. SHR6390 significantly blocks phosphorylation of Rb at serine 780 in six ESCC PDXs models. The levels of Rb, pRb were quantified by using the software Image Pro. *P < 0.05, **P < 0.01, ***P < 0.001, ****P < 0.0001 by one-way ANOVA or unpaired two-tailed t test

To characterize the underlying mechanisms observed in tumor growth inhibition, we measured the effects of SHR6390 in ESCC cells using western blots. As shown in Fig. 2a, SHR6390 reduced Rb phosphorylation in Eca 109 and KYSE-510 cell lines. However, SHR6390 failed to reduced Rb phosphorylation in Eca 9706 cell line which is the relative resistant to CDK4/6 inhibition. The in vitro results showed that pRb was significantly suppressed by effective treatment of SHR6390 in ESCC, which were also validated in vivo xenografts. We further examined the effects of SHR6390 in ESCC PDXs models using western blots. Consistent with in vitro results, SHR6390 reduced Rb phosphorylation in six PDXs models, and the decrease degree of pRb had some correlation with antitumor activity (Fig. 2b). This implies that the reduction in Rb phosphorylation is a generalized phenomenon occurring after CDK4/6 inhibition which might be the determinant of the therapy response.

### SHR6390 induced cell cycle arrest at the G1 phase rather than cell apoptosis

**Fig. 3:**
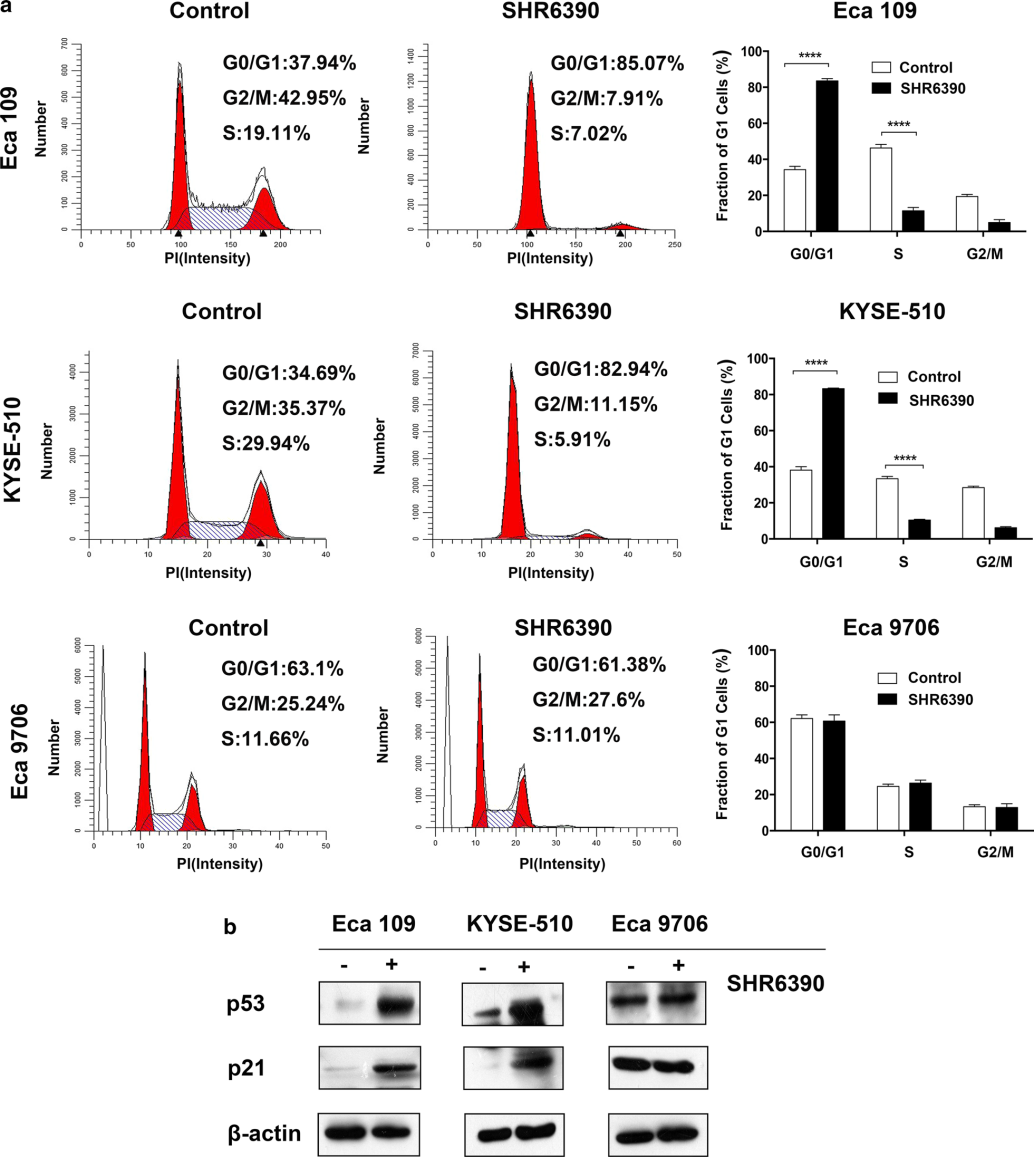
SHR6390 induced cell cycle arrest at G1 phase in Eca 109 and KYSE-510 cell lines. **a** Flow cytometric analysis showed that the percentage of cells accumulated in G1 phase significantly increased after treatment with SHR6390 alone compared to the control in Eca 109 and KYSE-510 cell lines not in Eca 9706 cell line. **b** The expressions of p53 and p21 was further upregulated by SHR6390 concomitant with cell cycle arrest at G1 phase. The data are expressed as the mean ± SD of three independent experiments. *P < 0.05, **P < 0.01, ***P < 0.001, ****P < 0.0001 by one-way ANOVA or unpaired two-tailed t test

To explore the potential mechanisms responsible for the inhibitory effect of SHR6390 in ESCC, cell cycle and cell apoptosis analyses were conducted in ESCC cell lines. Here we explored that SHR6390 treatment led to significant increases of G1 arrest and decreases in the S-phase fraction in Eca 109 and KYSE-510 cells, which was not found in Eca 9706 cells (Fig. 3a). Concomitant with the cell cycle arrest at the G1 phase, the expressions of cell cycle related proteins of p53 and p21 were upregulated after drug management in Eca 109 and KYSE-510 cells not in Eca 9706 cells (Fig. 3b). We also evaluated cell apoptosis in Eca 109 and Eca 9706 cell lines which responded differently to SHR6390. However, cell apoptosis was not induced by treatment with SHR6390 in Eca 109 and Eca 9706 cell lines (Additional file 1: Figure S1), which means SHR6390 inhibited ESCC cell proliferations mainly through cell cycle arrest.

### SHR6390 combined with PTX or CDDP offered synergistic inhibitory effects in ESCC xenografts

**Fig. 4:**
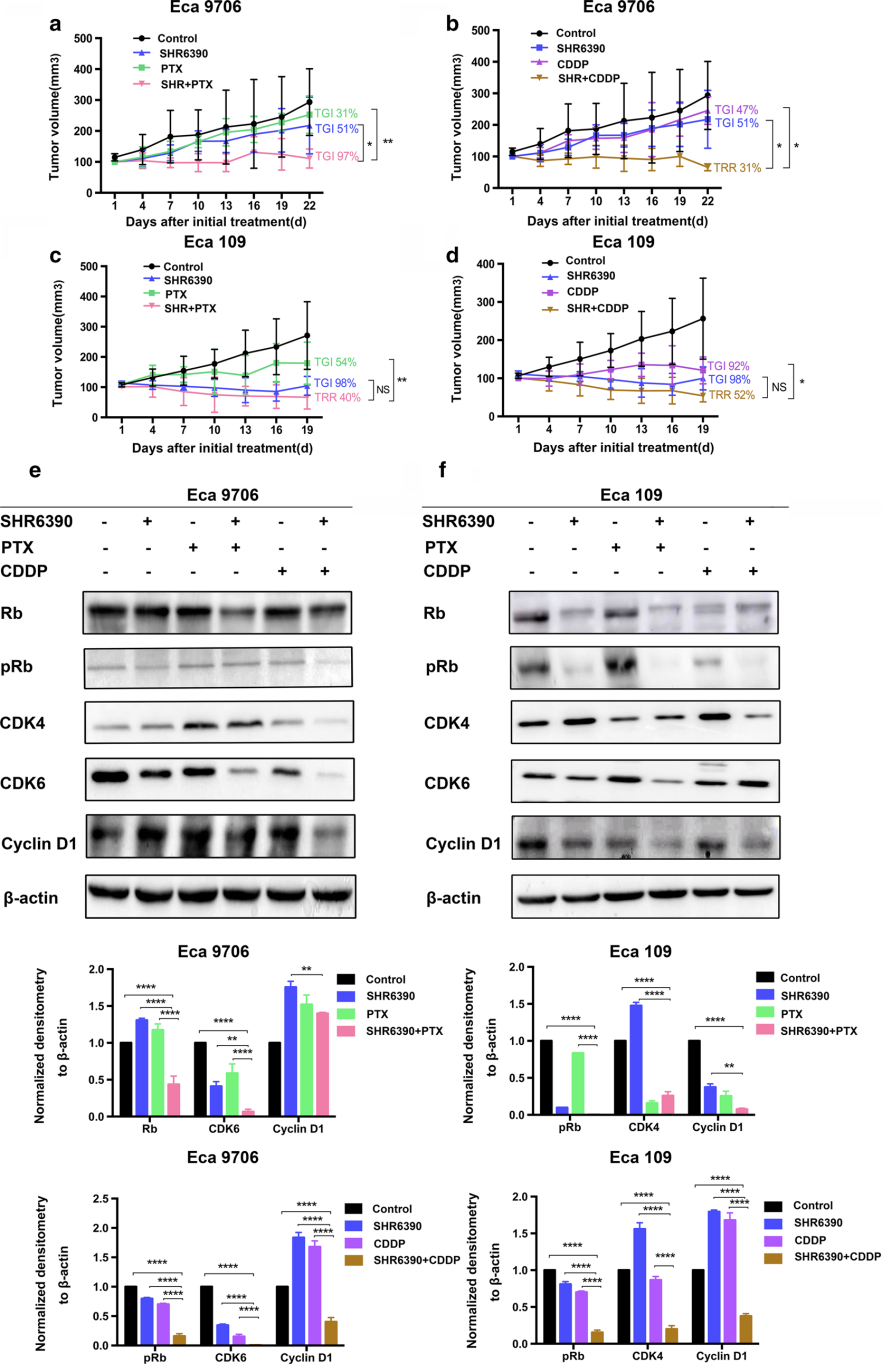
SHR6390 combined with PTX or CDDP offered synergistic inhibitory effects in ESCC xenografts. **a**–**d** Antitumor effects of SHR6390 and PTX or CDDP alone or in combination in Eca 9706 and Eca 109 xenografts. The growth of tumors treated with SHR6390 combined with PTX or CDDP was significantly suppressed in the Eca 9706 xenografts. **e**, **f** Effects of combination therapy on the Cyclin D1-CDK4/6-Rb pathway in vivo. Tumor specimen of Eca 109 and Eca 9706 xenografts were harvested after the end of treatment followed by analysis of lysates for Rb, pRb, CDK4, CDK6, Cyclin D1. The levels of Rb, pRb, CDK4, Cyclin D1 and CDK6 were quantified by using the software Image Pro. Tumor growth inhibition (TGI) values were calculated using the following formula: TGI = ΔT/ΔC × 100% (ΔT = tumor volume change of the drug-treated group, ΔC = tumor volume change of the control group on the final day of the study). Tumor regression rate (TRR) = (pre-drug tumor volume-post-drug tumor volume) × 100%/pre-drug tumor volume. Tumor volumes are expressed as mean ± SD. *P < 0.05, **P < 0.01, ***P < 0.001, ****P < 0.0001 by one-way ANOVA or unpaired two-tailed t test

Our results demonstrated clear potential of SHR6390 in ESCC. Nowadays, PTX and CDDP are chemotherapeutic agents which are most commonly used for the treatment of ESCC. We therefore evaluated the combination of SHR6390 with PTX or CDDP using Eca 109 and Eca 9706 xenografts in vivo. As shown in Fig. 4a, b, treatment with SHR6390 combined with PTX or CDDP showed synergistic inhibitory effects in Eca 9706 xenografts which showed relative lower sensitivity of SHR6390 single. SHR6390 also enhanced chemotherapy responses in Eca 109 xenograft, though combination therapy did not cause significantly changes compared to SHR6390 single therapy (Fig. 4c, d). Our results suggested that SHR6390 could enhance the chemotherapy sensitivity of ESCC in vivo, especially in cells which showed relative lower sensitivity of SHR6390 single, which will be validated in future studies.

To investigate the in vivo effects of combination therapy in CDK4/6 pathway, we compared the expressions of Rb, pRb, CDK4, CDK6 and Cyclin D1 between single treatment and combination reagents. CDK6 was downregulated in Eca 9706 xenografts with combination therapy than single therapy. Meanwhile, pRb and Cyclin D1 were downregulated in Eca 109 xenografts to a greater extent than either single therapy alone. These results suggested that, combined with PTX or CDDP, SHR6390 produced better synergy by regulating molecules in the cell cycle pathways.

### Identification of biomarkers associated with sensitivity to SHR6390

**Fig. 5:**
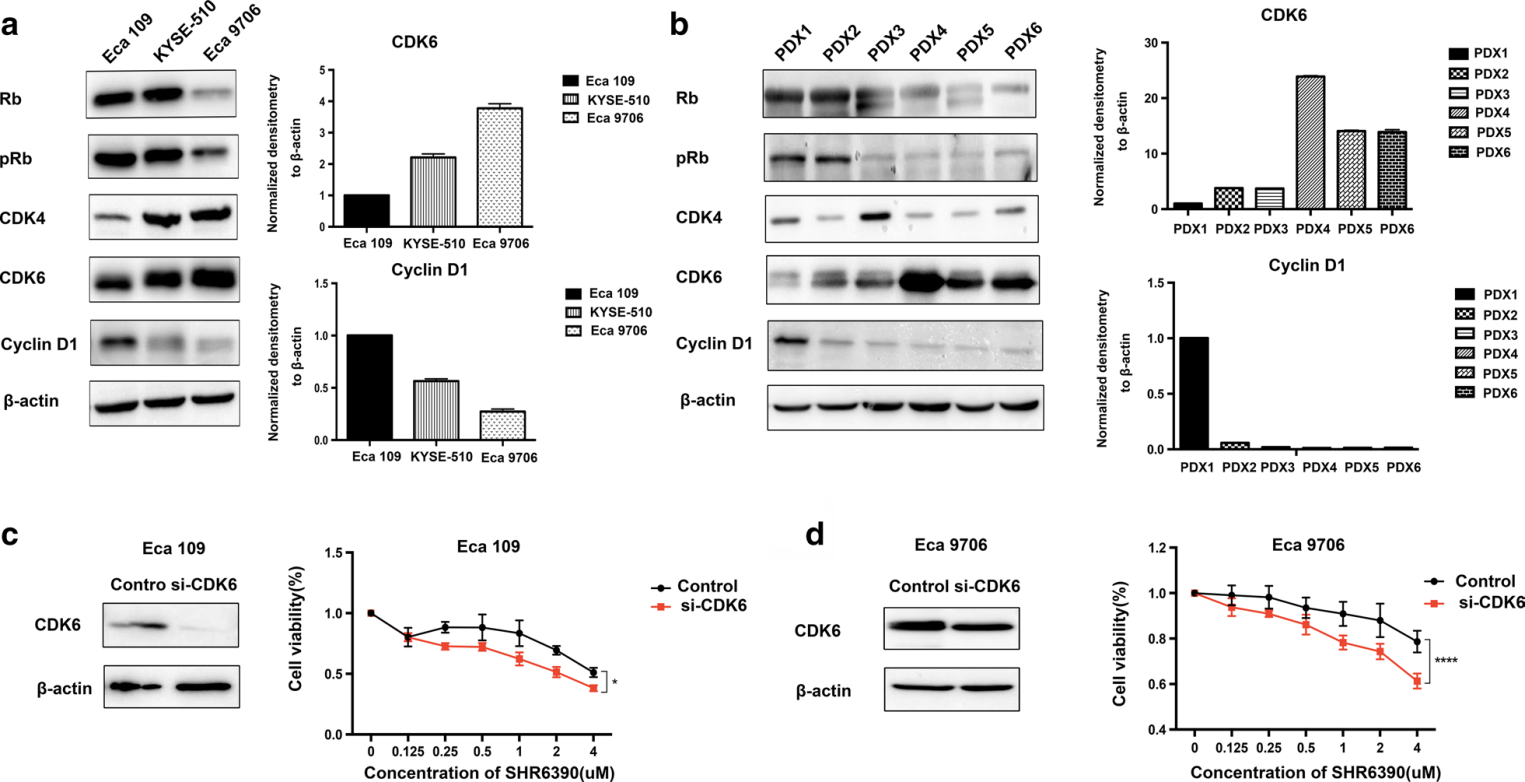
Identification of biomarkers associated with sensitivity to SHR6390. **a** Baseline expressions of CDK4/6 pathway related molecules in ESCC cell lines. The ESCC cell lines with high levels of CDK6 or low levels of Cyclin D1 expressions associated with reduced sensitivity to SHR6390. **b** Baseline expressions of CDK4/6 pathway related molecules in ESCC PDXs. The ESCC tumors untreated, from the same initial engraft were individually processed, and the levels of certain cell cycle proteins were analyzed. The PDXs with high levels of CDK6 or low levels of Cyclin D1 expressions associated with reduced sensitivity to SHR6390. **c**, **d** siRNA-mediated knockdown of CDK6 on CDK4/6 inhibition. Eca 109 and Eca 9706 cell lines were transfected with CDK6 siRNA or control siRNA. SHR6390 added 48 h after transfection. Cell proliferation was measured. After RNA interference against CDK6, Eca 109 and Eca 9706 cell lines showed an increased sensitivity to SHR6390

Our results demonstrated clear potential for SHR6390 single treatment or combined with chemotherapy in ESCC. However, different sensitivity to SHR6390 was observed in different cell lines or xenografts. To yield better response of SHR6390, we specifically explored the correlation between cell cycle biomarkers implicated in CDK4/6 signaling response to SHR6390. We assessed Cyclin D1-CDK4/6-Rb pathway related protein expressions in three cell lines and six PDXs models. The results showed that cell lines and PDXs with higher levels of CDK6 or lower levels of Cyclin D1 expressions might be associated with reduced sensitivity to SHR6390 (Fig. 5a, b).

To validate the association between CDK6 expression and response to SHR6390, we investigated whether downregulated protein level of CDK6 would alter the growth inhibitory effects of SHR6390 in Eca 109 and Eca 9706 cell lines. As shown in Fig. 5c, d, after RNA interference against CDK6, Eca 109 and Eca 9706 cell lines showed an increased sensitivity to SHR6390 with downregulated CDK6 expression. Also, we investigated whether downregulated protein level of Cyclin D1 would alter the growth inhibitory effects of SHR6390 in Eca 109 cell line. Our results showed that Eca 109 cell line with the observed induction of Cyclin D1 dramatically decreased the response to SHR6390 (Additional file 2: Figure S2). Collectively, our data indicated that low expression of CDK6 and/or high expression of Cyclin D1 were correlated with the high sensitivity of SHR6390 in ESCC, which would be helpful to select patients benefit from SHR6390.

## Discussion

The antitumor activity of CDK4/6 inhibitor were observed in many preclinical studies and the encouraging results from these studies have prompted clinicians to evaluate its efficacy and safety in clinical trials. In a single-arm study, five of 17 patients with relapsed mantle cell lymphoma remained progression-free for more than 1 year on CDK4/6 inhibitor therapy, with one complete response (CR) and two partial responses (PRs) [16]. The phase 2 study PALOMA-1 involved 165 postmenopausal women with advanced ER-positive/HER2-negative breast cancer who had not received any systemic treatment for their advanced disease. All patients were randomly assigned 1:1 to receive letrozole or letrozole plus CDK4/6 inhibitor. After a median follow-up of 29 months, median PFS was 20.2 months for the CDK4/6 inhibitor combined with letrozole group and 10.2 months for the letrozole group [9]. However, the role of CDK4/6 inhibitor in treating ESCC is still unclear.

Given the presence of highly mutation loads in Cyclin D1-CDK4/6-Rb signalling pathway-related molecules of ESCC [9–11], we investigated the effect of CDK4/6 inhibitor SHR6390 in our study. Treatment of SHR6390 suppressed cell proliferation and tumor growth in ESCC cell lines and xenografts by inhibiting levels of pRb and effectively arrested the cell cycle at the G1 phase with the expression levels of p53 and p21 were upregulated. Hypophosphorylation of Rb due to CDK4/6 inhibition lead to the inhibition of E2F activity which is crucial for DNA synthesis and progression of cell cycle [17]. Li et al. reported that CDK4/6 inhibitor treatment induces cell cycle arrest at the G1 phase and thus suppresses the proliferation of colorectal carcinoma cells [18], which was similar to our results. p21 is one of the CDK inhibitor, which considered as the most potent regulator of cell cycle. It involved in blocking G1 cell cycle progression, which is controlled in a tumor suppressor p53-dependent manner [19]. On the other hand, SHR6390 treatment failed to induce significant apoptosis in Eca 109 cells, dedicating that SHR6390 inhibited tumor growth mainly through arresting cell cycle at G1 phase.

Our data demonstrated that combination therapy yield greater anti-tumor activity than individual treatment in Eca 9706 xenografts. It has been reported that CDDP could induce DNA damage-mediated cytotoxicity in cells that are arrested in the G1 phase [20]. Thus, SHR6390 mediated G1 accumulation of cells may sensitize them to CDDP. Also, Zhang et al. reported that combined CDK4/6 inhibition and paclitaxel produced synergistic antitumor activity and increased apoptosis through reduced Cyclin D1 and Bcl-2 in cancer cells [21]. Modeling studies indicate that combinations of effective cytostatic and cytotoxic drugs should increase cure rates by delaying drug resistance and preventing tumor growth between treatments with cytotoxic agents [5]. However, CDK4/6 inhibitors combined with standard cytotoxic chemotherapy showed conflicting results in preclinical studies. It has been reported that dual inhibition of CDK4/6 inhibitor and gemcitabine enhanced the antitumor effect in a xenograft model of lung cancer [22] and CDK4/6 inhibition also sensitized neuroblastoma cells to doxorubicin-induced apoptosis [23]. In contrast, CDK4/6 inhibitor reduced the cytotoxicity of antimitotic and platinum agents in preclinical models [24–26]. In future, we will optimize the combination regimen and search for other possible targets in pathways of PI3K-mTOR [27, 28] or MEK-ERK [10, 29], which may enhance the efficacy of CDK4/6 blockade.

The combination of CDK4/6 inhibitors and other therapy is being used in the clinic in unselected patient populations, but not all patients will benefit from such therapy [9]. Thus, there is an urgent need to identify biomarkers that predict response to CDK4/6 inhibitor. Preclinical studies have defined a series of biomarkers that respond to CDK4/6 inhibitors, of which the Cyclin D1-CDK4/6-Rb pathway has been the best one till now [30, 31]. Alterations in the expression of genes that related to the cell cycle are important in determining drug sensitivity to anticancer agents. We therefore sought to validate biomarkers that predict in vitro response to SHR6390 in cell cycle signaling. Our results show that low expression of CDK6 may correlated with high sensitivity of SHR6390. Similarly, Yang et al. reported that knockdown of CDK6 restored CDK4/6 inhibitor sensitivity, while enforced overexpression of CDK6 was sufficient to mediate drug resistance in breast cell lines. High CDK6 expression may affect response to CDK4/6 inhibition by preferentially binding to Cyclin D3, creating a resistant complex than the Cyclin D1-CDK4 complex [30]. Meanwhile, our results showed that higher expression of Cyclin D1 was correlated with SHR6390 higher sensitivity. However, in the PALOMA-1 study, patient selection based on Cyclin D1 amplification was not correlated with better outcome [9]. In addition to Cyclin D1 and CDK6 status, CDK4 has been proposed as a predictive biomarker of response to CDK4/6 inhibition [31]. However, our results indicated that level of CDK4 expression has no relationship with the sensitivity to SHR6390 (data not shown). Also, Rb status has been proposed as a selective biomarker of CDK4/6 inhibitor utility [17]. It has been reported that the efficacy of CDK4/6 inhibitors requires functional Rb expressing in tumor cells. However, variable growth inhibition among Rb-proficient cell lines and PDXs models suggests the existence of other factors that influence tumor cell sensitivity to SHR6390.

## Conclusions

In conclusion, our findings support further clinical evaluation of SHR6390 as a single agent or in combination with chemotherapy in ESCC patients. Moreover, the assessment of CDK6 and Cyclin D1 expressions may help to identify the patient subgroup most likely to benefit from treatment with SHR6390.

## Additional files

**Additional file 1: Figure S1.** Effects of SHR6390 on apoptosis in ESCC cell lines. **A**, **B** Annexin-V/PE-7ADD staining and flow cytometry data. We evaluated cell apoptosis in Eca 109 and Eca 9706 cell lines which responded differently to SHR6390. While SHR6390 did not cause significant apoptosis in Eca 109 and Eca 9706. **C** Effects of SHR6390 on apoptotic markers via Western blots. Cell apoptosis related protein Cas 8, Cas 3, Bcl-2 was not significantly changed by treatment with SHR6390.

**Additional file 2: Figure S2.** siRNA-mediated knockdown of Cyclin D1 on CDK4/6 inhibition. Eca 109 cells transfected with Cyclin D1 siRNA, control siRNA and then treated with SHR6390 48 h after transfection. Cell proliferation was measured. After RNA interference against Cyclin D1, Eca 109 cell line showed a reduced sensitivity to SHR6390.

## Abbreviations

ESCC: esophageal squamous cell carcinoma
TRR: tumor regression rate
TGI: tumor growth inhibition
PTX: paclitaxel
CDDP: cisplatin
