# Supplementary figures and images for: CDK4/6 inhibitor-SHR6390 exerts potent antitumor activity in esophageal squamous cell carcinoma by inhibiting phosphorylated Rb and inducing G1 cell cycle arrest
Source: J Transl Med. 2017 Jun 2;15:127. doi: 10.1186/s12967-017-1231-7 (PMC5457542; doi:10.1186/s12967-017-1231-7)

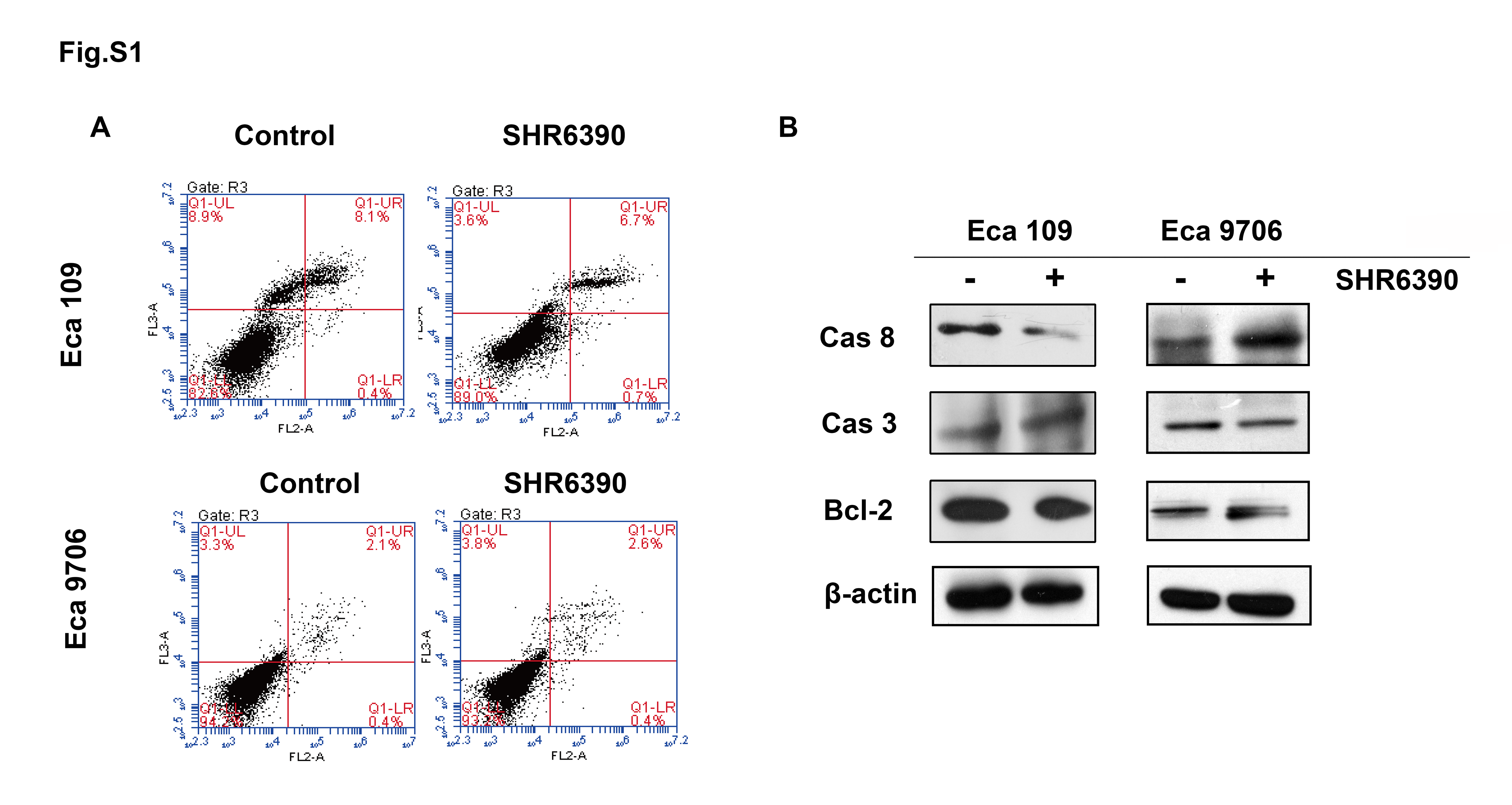

Supplement: Supplementary file 1 — Additional file 1: Figure S1. Effects of SHR6390 on apoptosis in ESCC cell lines. A, B Annexin-V/PE-7ADD staining and flow cytometry data. We evaluated cell apoptosis in Eca 109 and Eca 9706 cell lines which responded differently to SHR6390. While SHR6390 did not cause significant apoptosis in Eca 109 and Eca 9706. C Effects of SHR6390 on apoptotic markers via Western blots. Cell apoptosis related protein Cas 8, Cas 3, Bcl-2 was not significantly changed by treatment with SHR6390. [file 12967_2017_1231_MOESM1_ESM.tif]

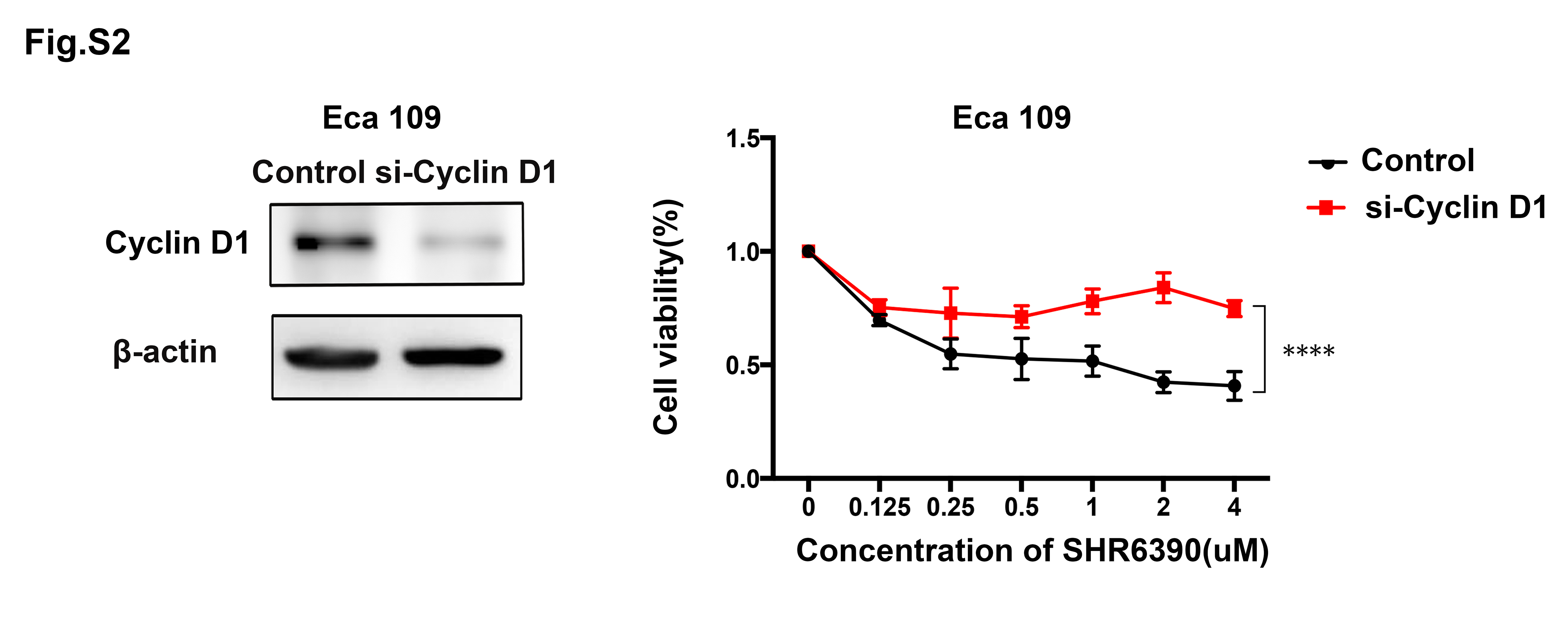

Supplement: Supplementary file 2 — Additional file 2: Figure S2. siRNA-mediated knockdown of Cyclin D1 on CDK4/6 inhibition. Eca 109 cells transfected with Cyclin D1 siRNA, control siRNA and then treated with SHR6390 48 h after transfection. Cell proliferation was measured. After RNA interference against Cyclin D1, Eca 109 cell line showed a reduced sensitivity to SHR6390. [file 12967_2017_1231_MOESM2_ESM.tif]
